# Impact of local hyaluronic acid on early gingival wound healing and heat shock protein 47

**DOI:** 10.1590/1678-7765-2026-0091

**Published:** 2026-07-27

**Authors:** Gizem Ceren Yiğit, Feyza Otan Özden, Özgür Korhan Tunçel, Eser Çakmak, Fikret Yilmaz

**Affiliations:** 1 Ondokuz Mayıs University School of Dentistry Department of Periodontology Samsun Turkey Ondokuz Mayıs University, School of Dentistry, Department of Periodontology, Samsun, Turkey; 2 Ondokuz Mayıs University School of Medicine Department of Biochemistry Samsun Turkey Ondokuz Mayıs University, School of Medicine, Department of Biochemistry, Samsun, Turkey; 3 Ondokuz Mayıs University School of Dentistry Department of Restorative Dentistry Samsun Turkey Ondokuz Mayıs University, School of Dentistry, Department of Restorative Dentistry, Samsun, Turkey

**Keywords:** Hyaluronic acid, Heat shock protein 47, Wound healing, Gingiva

## Abstract

Hyaluronic acid (HA) is important for tissue repair, especially in oral soft tissue healing.

**Objective:**

This study aimed to evaluate the effect of HA on gingival healing and its relationship with heat shock protein 47 (HSP 47), which binds to procollagen.

**Methodology:**

40 female Wistar albino rats were divided into two groups: a control (Group C) and an HA-treated group (Group H), each split into four subgroups for different time points (t1, t3, t7, t14 days). Excisional wounds were made in the palates of all rats. Group H received topical HA gel, whereas Group C received no treatment. HSP 47 levels were measured using enzyme-linked immunosorbent assay, and wound areas were analyzed with ImageJ.

**Results:**

No significant difference in HSP 47 levels was observed between groups (p>0.05). However, wound areas in Group C were significantly larger than in Group H on day 3 (p=0.009). HSP 47 levels increased in both groups, with higher values on day 1 (p=0.043). Wound areas decreased over time in both groups, with significant reduction in Group H from days 1 to 3 (p=0.043).

**Conclusion:**

Topical HA gel may accelerate early periodontal wound healing but failed to significantly affect HSP 47 levels.

## Graphical abstract

**Figure:**
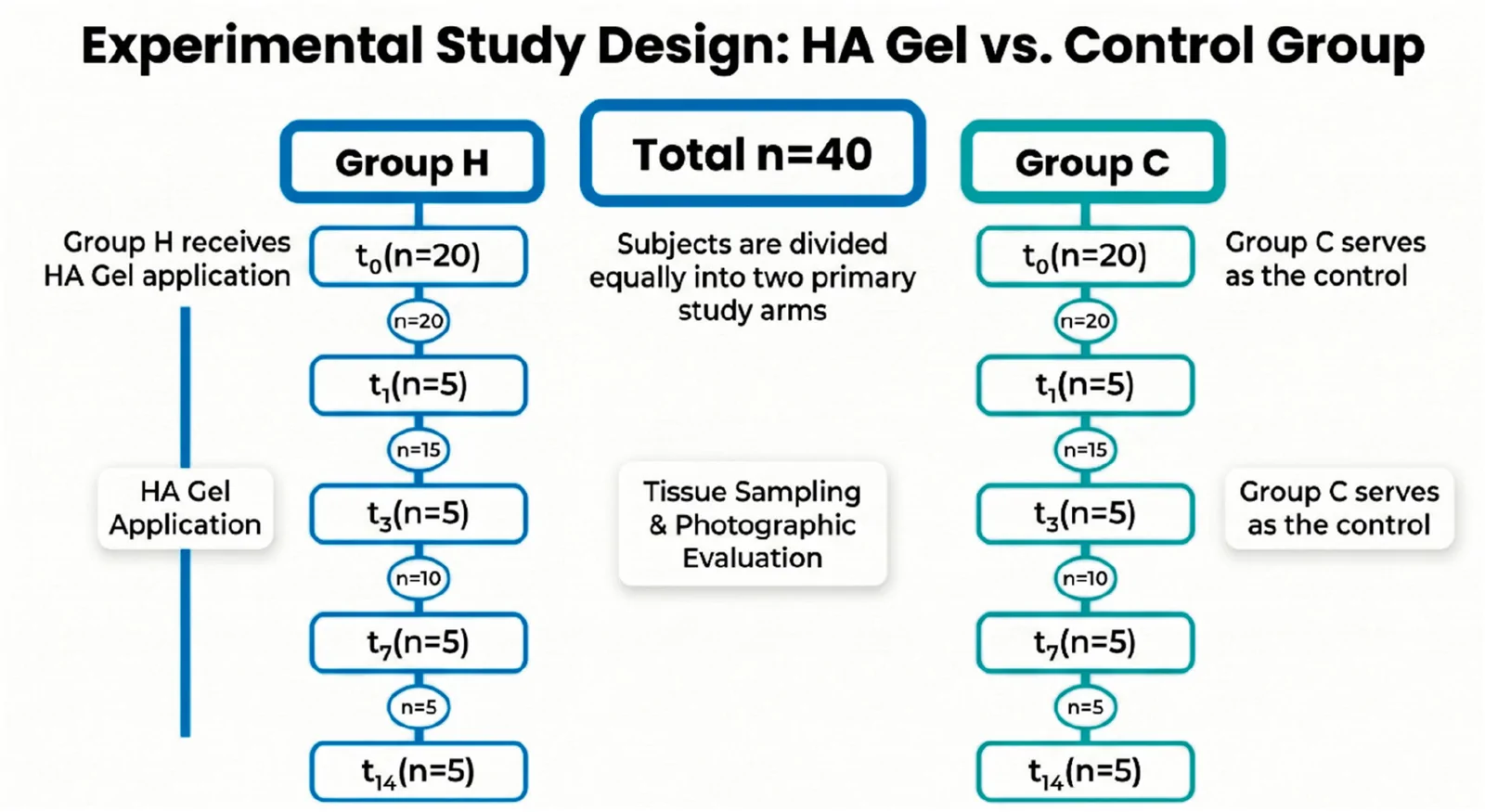

## INTRODUCTION

Wound healing occurs as physiological turnover in periodontal tissues and ensures tissue continuity after periodontal treatments. Various agents and treatment methods (such as laser therapy, hyperbaric oxygen therapy, and herbal preparations) have been used to accelerate periodontal wound healing for reducing the possibility of infection and patient discomfort.^1-4^

Hyaluronic acid (HA), an agent thought to have positive contributions to wound healing, has recently started to be used in dentistry. Positive effects of exogenous HA agents on tissue repair and accelerated wound healing have been shown in clinical studies.^5^ HA, a polymer that is a protype of the glycosaminoglycan family, is found in all living organisms, especially in the extracellular matrix of connective tissue. It plays a dynamic role in biological events such as cell migration and morphogenesis during tissue repair and regeneration.^6,7^ Functions of HA in slowing down the effects of inflammation in wound healing stages and supporting cell proliferation have been shown.^8^ The biological effects of HA are influenced by its molecular weight. High molecular weight HA is generally associated with anti-inflammatory and immunosuppressive effects, whereas low molecular weight HA fragments may promote inflammatory responses by stimulating cytokine production. Therefore, HA may exert dual roles during wound healing depending on its molecular characteristics and local microenvironment.^9^

It has been reported that HA supports the inflammatory response, especially in periodontal wound healing, and ultimately induces the production of proinflammatory cytokines by endothelial cells, fibroblasts, keratinocytes, cementoblasts, and osteoblasts.^10^ HA preparations have been reported to increase the migration and proliferation ability of fibroblasts and contribute to soft tissue healing/regeneration after regenerative periodontal surgery.^10,11^ Antiedema, anti-inflammatory, and wound healing properties have been reported after the exogenous application of HA.^12^

Heat shock proteins (HSPs), proteins with several intracellular functions, are secreted from cell organelles in response to stress factors such as temperature increase, ischemia, hypoxia, and tissue trauma. They are found in all organisms. HSPs maintain cellular balance under physiological conditions and prevent cell damage in stressful situations. Synthesized HSPs have broad substrate properties, whereas HSP 47, a member of the HSP family, specifically binds to procollagen. HSP 47 is an endoplasmic reticulum-resident molecular chaperone required for the correct folding of procollagen in the endoplasmic reticulum.^13,14^ HSP 47 binds to the triple helix procollagen form and stabilizes it in the endoplasmic reticulum, thus ensuring the efficient transport of procollagen from it to the Golgi apparatus. Expression of HSP 47 is closely associated with collagen expression in various tissues and cell types and is observed in collagen-producing cells such as fibroblasts.^13^ In healthy gingiva, 99% of the extractable collagen is type I and III, whereas less than 1% is type V.^15^ Relative mRNA expression levels of collagen (types I, III, and V) have been reported to be released when levels of HSP 47 were upregulated in the scleral fibroblasts of guinea pigs. This indicates that HSP 47 can promote the synthesis of collagen.^16^ Given its central role in collagen biosynthesis, HA likely exerts its effects on fibroblast function by directly or indirectly regulating HSP47 expression or activity.^17^ If HA upregulates HSP47 in fibroblasts or other skin-resident cells, it may explain how HA accelerates organized collagen deposition and scar maturation. Investigating this potential regulatory pathway may reveal new mechanistic insights into how HA enhances wound healing at the molecular level in gingival wound healing.

Although the action of HA on the production of collagens by endothelial cells have been proved during wound healing, to date no study has examined its effect on HSP 47 expression. Therefore, this study aimed to show for the first time the effect of HA on gingival wound healing by examining the wound surface area and HSP 47 release.

## Methodology

### Experimental design

All experimental procedures were approved by the Ethics Committee for Animal Experimentation at Ondokuz Mayıs University (approval number: 2019/07). They were performed according to the mandatory regulations of this committee. All experimental procedures were performed at the Ondokuz Mayıs University Laboratory of Experimental Animals Research Center. The biochemical analysis was carried out in Ondokuz Mayıs University Faculty of Medicine Department of Medical Biochemistry.

In this study, 40 adult (aged six-seven weeks) female Wistar rats (≈125 to 150 g) were housed with food and water *ad libitum* at constant room temperature (22±1°C) under a 12-h light/dark cycle in individual cages. The animals were randomly divided into two equal groups: the control (Group C) and HA (Group H) groups. Initial tissue sampling for baseline measurements and photographic evaluations was performed without additional animal sacrifice, ensuring that the total number of animals (n=20) was sufficient for all planned assessments. After baseline tissue sampling and photographic evaluation, each group was randomly divided into four subgroups consisting of five animals according to the experimental days: days 1 (t1, n=5), 3 (t3, n=5), 7 (t7, n=5), and 14 (t14, n=5). Tissue samples and photographic images (Figure 5) were continued to be obtained from five animals from each group on the following experimental days (t1, t3, t7, t14). In each period, five animals were excluded from the groups.

The flow of the study is summarized in Figure 1.

**Figure 1:**
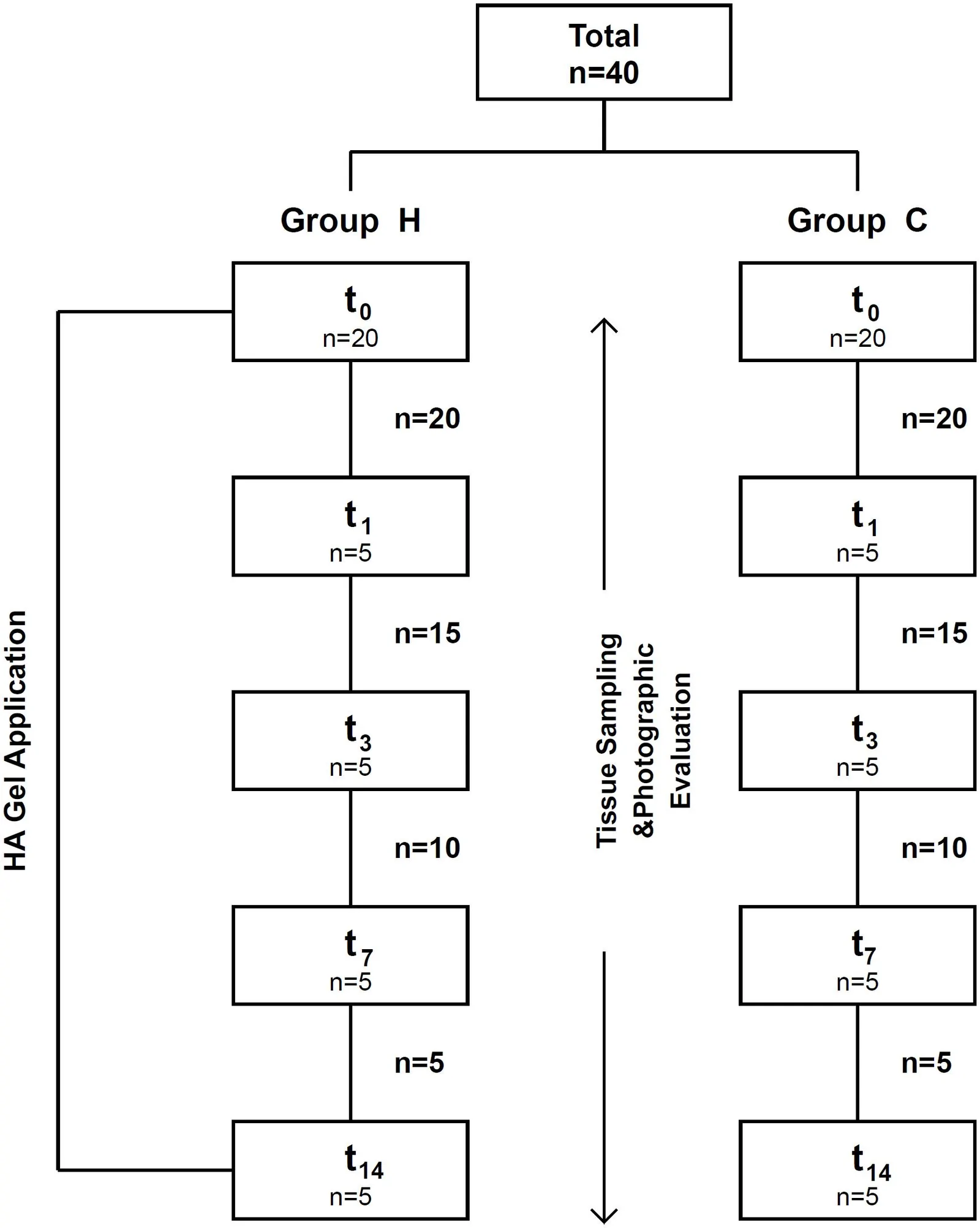
The flow of the study.

### Surgical procedures

First, 50 mg/kg ketamine and 10 mg xylazine were intraperitoneally given in combination to all rats to provide systemic anesthesia. As part of a standardized procedure, soft tissue with a 4-mm diameter was removed from the palatal region using supraperiosteal punctures, which is commonly employed in experimental models for consistent wound creation.^18^ Tissue samples were marked as baseline (t0) according to the group and rat number and placed in 2-ml capped storage tubes (Eppendorf AG, Hamburg, Germany) to be stored in an ultralow freezer (NuAire, No: 9394248) at −80°C until the biochemical analysis.

The surgical and anesthetic procedures performed under the same conditions were repeated during the tissue uptake and HA administration throughout the experiment. No animals were sacrificed in this study.

### Application of the HA gel

The HA gel (0.8%) in this study is a commercially available non-animal origin gel (hyaDENT BG, BioScience GmbH, Germany); 1 ml of it contains 2.0 mg of non-cross-linked HA, 16.0 mg of cross-linked HA, 6.9 mg of sodium chloride, and 1.0 ml of water to make it injectable with the molecular weight of 1 million Dalton. First, 10 µl of 2% g/ml HA gel was applied to the wound in the palatal region in Group H for 30 seconds via sterile cannulas. Local HA application was continued at 24-hour intervals, with five animals being removed on each experimental day.

The animals in the control group were subjected to no procedure except tissue sampling and photographing up to the end of the experimental period.

### Wound area measurement (WAM)

The wounds were photographed to enable macroscopic evaluation and comparisons between groups on the experiment days. The photos were taken with the same camera (Nikon D7000 – Tokyo, Japan, 105-mm lens) from the appropriate distance and by the same researcher (GCY) to follow a set standard. The photographs were taken in large JPEG and RAW formats using a ring flash (Metz 15 MS-15 digital) with manual focus at ISO: 125, white balance flash, 1/100 shutter speed, and F36 aperture. They were transferred onto ImageJ (National Institutes of Health, Bethesda, MD, USA). The software was calibrated with a Williams periodontal probe (Hu-Friedy, Chicago, IL, USA). It was positioned in the area during photographing, and the wound area was calculated in square millimeters by measuring the pixels in the area via the software (Figure 2). All surgical procedures and surface measurements were made by the same investigator (ÖKT), who was blinded regarding the study design. Area measurements were performed three times, and the mean value was calculated.

**Figure 2:**
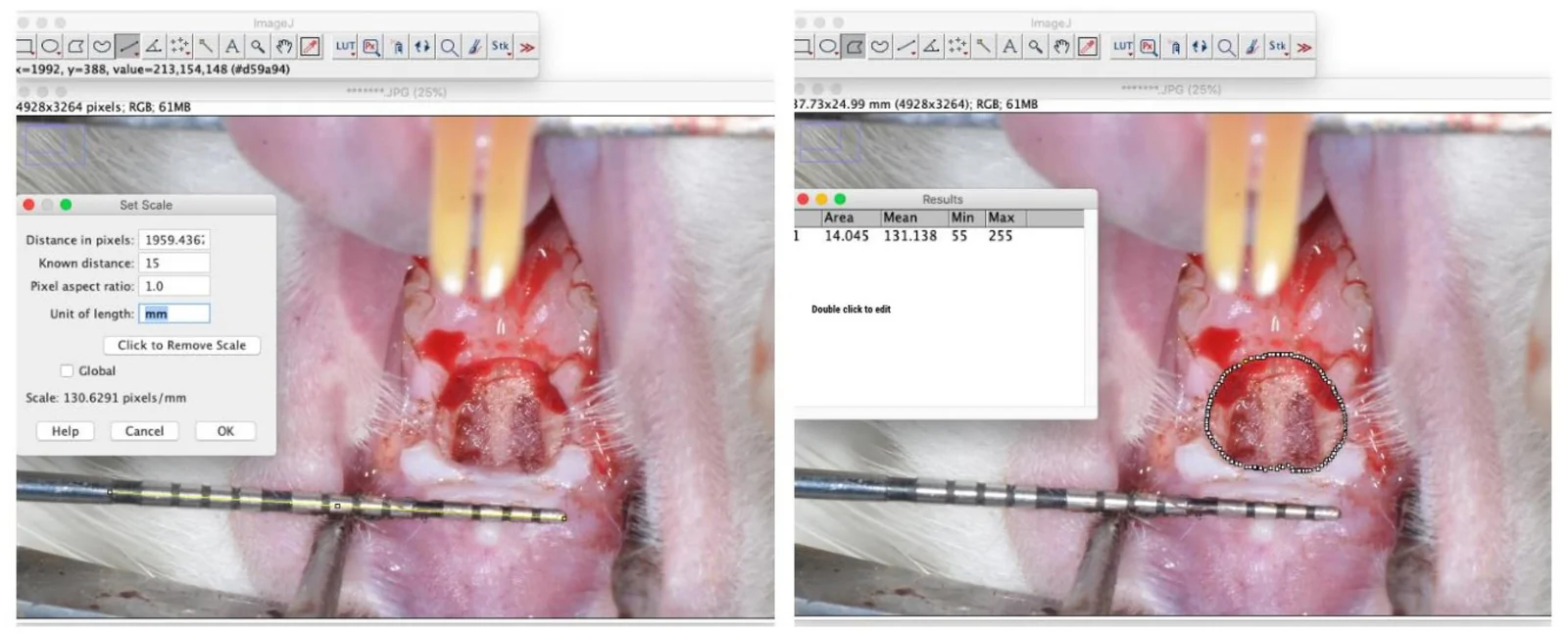
(**a**) Calibration of the periodontal probe in the photograph to its actual size (**b**) marking of the wound site and calculation of the surface area.

### Analysis of HSP 47

Tissue samples in Eppendorf tubes were kept in an ultralow freezer at −80°C. The tissues were cleaned of blood by adding 1 ml of deionized water and sonicating them at 220 volts at +4°C for about 15-20 seconds (Fisher, Sonic Dismembrator; Model 300). Each sample was then crushed and homogenized in a mortar using liquid nitrogen. The homogenized tissue was added to a buffer containing 1 ml of pH 7.4, 0.01 M phosphate-buffered serum and sonicated again for 1 minute at 220 volts at +4°C to ensure precise homogenization. The samples were centrifuged at 3000 × *g* for 20 minutes (Sigma, Laboratory Centrifuges; 3K30). The supernatants were used for biochemical analysis.

HSP 47 protein levels were determined using the commercially available YL Biont Rat Heat Shock Protein (HSP 47) ELISA Kit^®^ (YL Biont, Cat. No: YLA1007RA, Shanghai, P.R. China). The procedures were carried out in line with commercial company directives. The sensitivity of this kit totals 3.52 ng/L; its measuring range, 10 ng/L-1000 ng/L; its intra-assay CV, <8%; and its inter-assay CV, <10%. HSP47 concentration was subsequently normalized to the total protein content of each sample, as determined by the Lowry method.^19^ Therefore, HSP47 levels are shown as ng/mg protein to enable standardized comparison between specimens.

### Statistical analysis

In calculating the number of samples, the number of animals in the groups was determined based on a limited number of similar studies (similar experimental periods; one, three, seven, 14 days) in which a wound was created in the palatal area and wound healing was performed by removing rats from the experimental group for the specified periods.^27,36^ The total sample size was determined using repeated measures ANOVA, assuming a 0.25 effect size, a 0.05 margin of error, and a 0.95 statistical power. Accordingly, the required sample size was calculated as 36. Considering a potential data loss of approximately 10%, the total number of experimental units was increased to 40. Moreover, an a priori power analysis (power = 0.99, α = 0.05) was conducted to assess sample size adequacy. Based on a two-sample t-test power analysis, it was determined that at least five rats per group would be sufficient to achieve 99% power with a 5% type I error rate. The data were evaluated on IBM SPSS Statistics 23 and checked for normality by the Shapiro–Wilk test. As the data were not normally distributed, non-parametric tests were applied. Intergroup comparisons were performed using the Mann–Whitney U test, and intragroup comparisons were conducted using the Wilcoxon signed-rank test. Measures of central tendency (median, IQR, minimum, and maximum) were used for numerical variables. Statistical significance was set at p ≤ 0.05 level for all tests.

## RESULTS

### Intergroup analyses

Values for the distribution of HSP 47 and WAM are given in Table 1. Groups C and H showed no statistically significant difference regarding HSP 47 values at baseline (t0) or on the other days of the experiment (t1, t3, t7, t14) (p>0.05). Changes in the HSP 47 values and the difference between the groups are shown in Figure 3 and Table 2.

**Table 1:** Distribution of HSP 47 and WAM values.

	Group C	Group H
**t**_0_ **HSP 47**	0.36/0.21(0.2-7.2)	0.26/0.13(0-1.7)
**t**_1_ **HSP 47**	3.47/1.91(0.2-4.2)	0.27/1.79(0.2-2.9)
**t**_3_ **HSP 47**	0.23/0.09(0.1-0.3)	0.16/0.07(0.1-0.3)
**t**_7_ **HSP 47**	0.17/0.06(0.1-0.6)	0.13/0.02(0.1-0.2)
**t**_14_ **HSP 47**	0.18/0.17(0.1-0.3)	0.18/0.09(0.1-0.5)
**t**_0_ **WAM**	12.18/0.81(11.7-13.4)	12.61/0.76(10.5-13.5)
**t**_1_ **WAM**	10/0.13(9.8-10.5)	9.46/0.66(8.1-10.2)
**t**_3_ **WAM**	10.34/0.56(9.2-10.9)	7.82/0.82(6.9-8.7)
**t**_7_ **WAM**	1.84/0.24(1.3-3.7)	1.64/0.85(1.2-2.9)
**t**_14_ **WAM**	0.42/1.13(0.2-1.5)	0.05/0.21(0-1.2)

Note: Values reported as medians/IQRs (minimum - maximum).

Abbreviations: HSP 47, Heat Shock Protein 47; Wound Area Measurement, WAM; Group C, control group; Group H, hyaluronic acid group; t0, baseline; t1, day 1; t3, day 3; t7, day 7; t14, day 14.

**Table 2:** Intergroup comparisons of HSP 47 values.

	Group C	Group H	Test/p
**t**_0_ **HSP 47**	0.36/0.21(0.2−7.2)	0.26/0.13(0−1.7)	Z0= −1.515 p=0.130
**t**_1_ **HSP 47**	3.47/1.91(0.2−4.2)	0.27/1.79(0.2−2.9)	Z1= −1.358 p=0.175
**t**_3_ **HSP 47**	0.23/0.09(0.1−0.3)	0.16/0.07(0.1−0.3)	Z3= −1.149 p=0.251
**t**_7_ **HSP 47**	0.17/0.06(0.1−0.6)	0.13/0.02(0.1−0.2)	Z7= −0.522 p=0.602
**t**_14_ **HSP 47**	0.18/0.17(0.1−0.3)	0.18/0.09(0.1−0.5)	Z14= −0.104 p=0.917

Note: Values reported as medians/IQRs (minimum and maximum). Data were statistically analyzed by the Mann–Whitney U test. Statistical significance was set at p ≤ 0.05.

Abbreviations: HSP 47, Heat Shock Protein 47; Group C, control group; Group H, hyaluronic acid group; t0, baseline; t1, day 1; t3, day 3; t7, day 7; t14, day 14.

**Figure 3:**
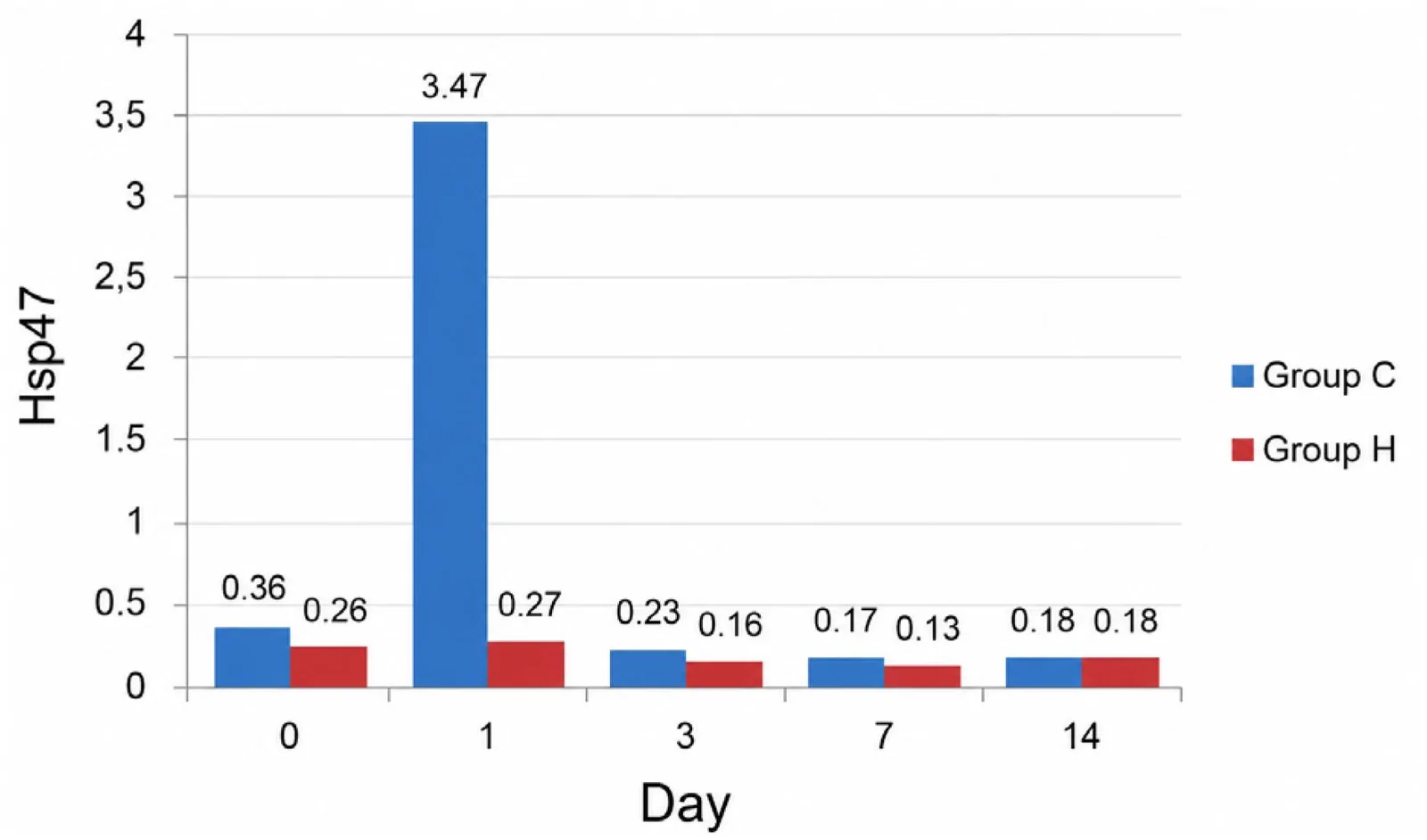
Distribution of HSP 47 between the groups on the test days.

WAM values showed a statistically significant difference between the groups at t3 (p<0.05). WAM values in Group C were significantly higher than those in Group H, indicating that Group H showed more healing in the wound area than Group C at t3 (Figure 4, Figure 5, and Table 3).

**Figure 4:**
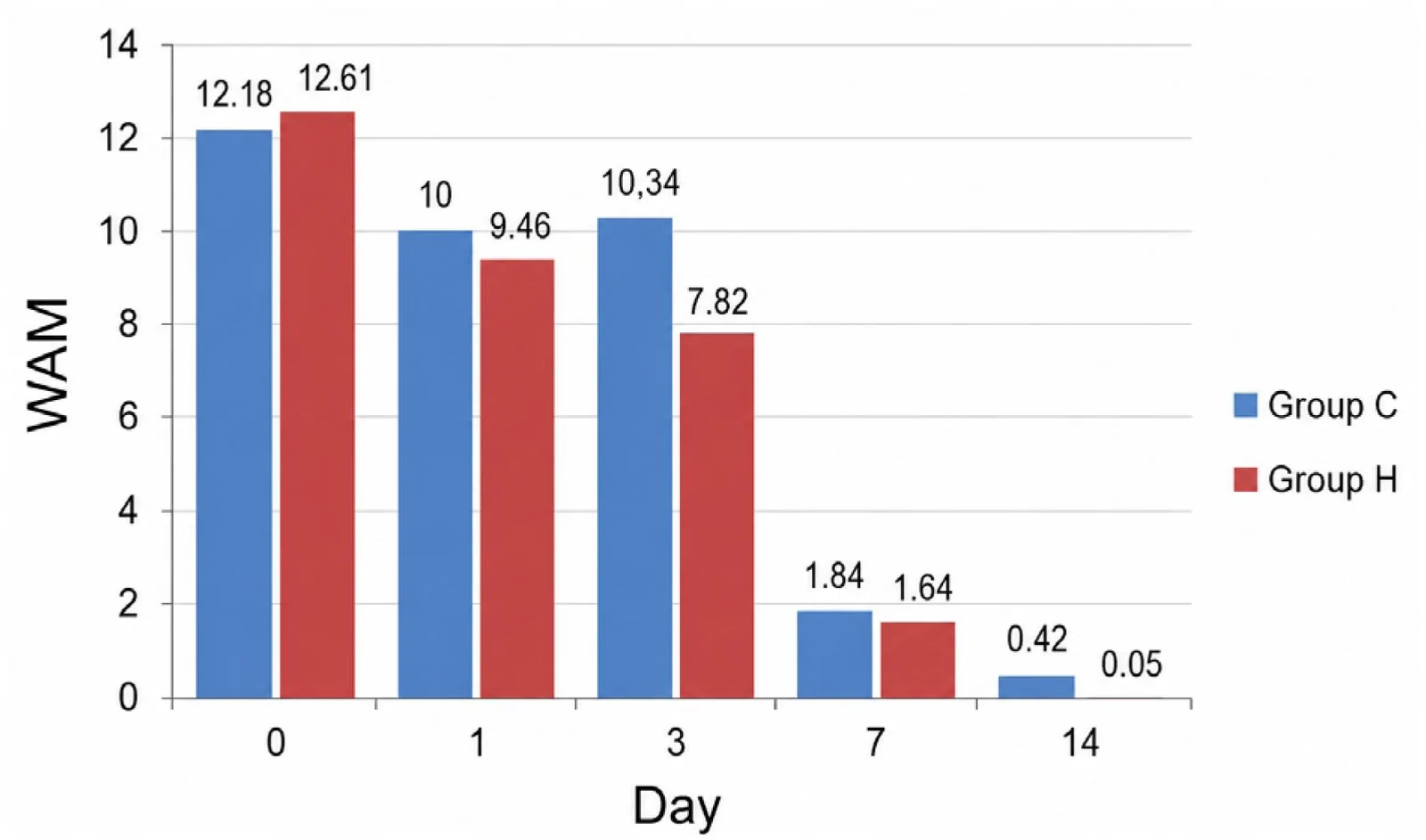
Distribution of WAM between the groups on the test days.

**Figure 5:**
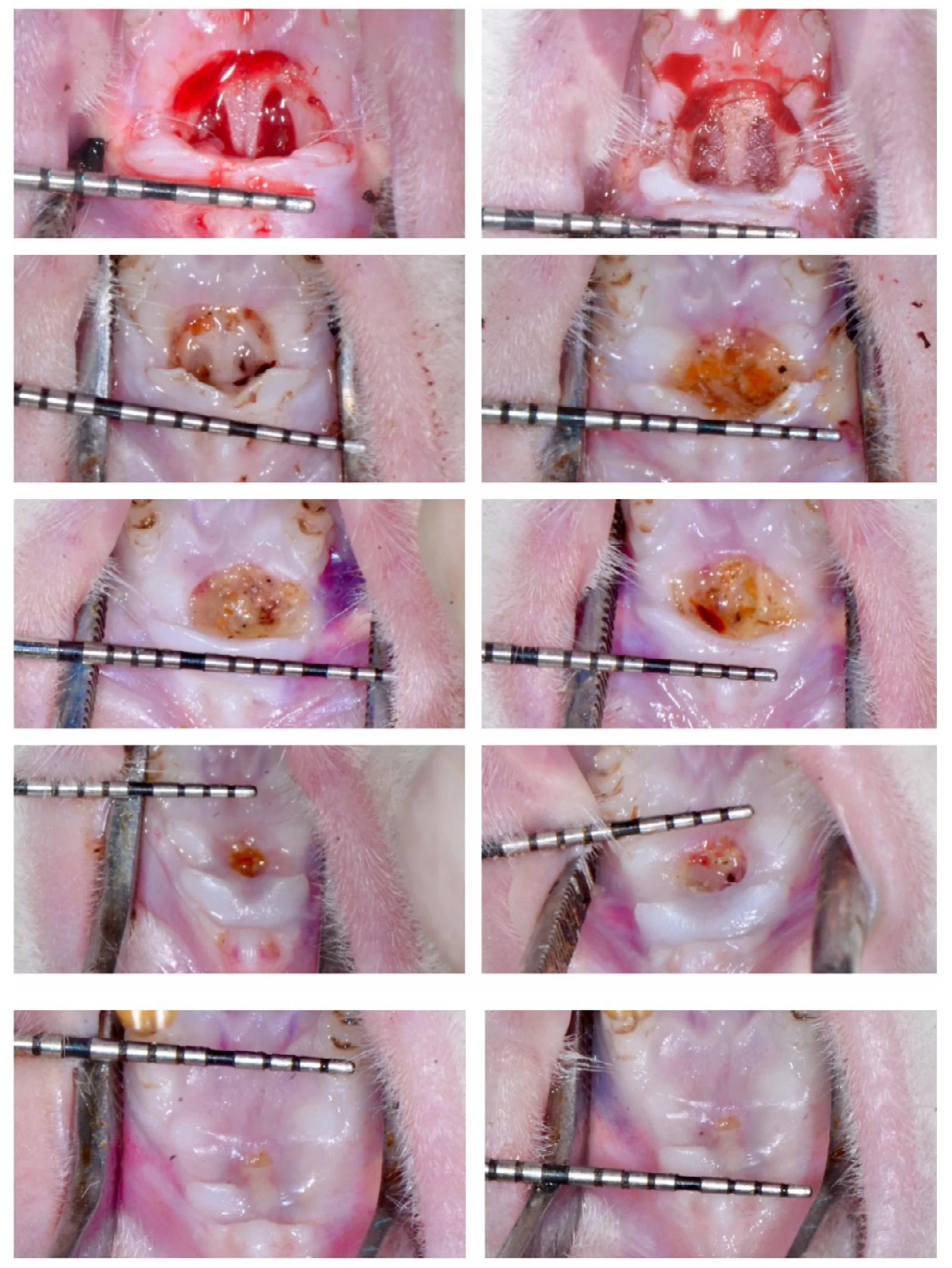
Clinical comparison for WAM: The first day of the experiment for Group C and Group H (**a, b**), wound healing on day 1 in Group C and Group H (**c, d**), wound healing on day 3 in Group C and Group H (**e, f**), wound healing on day 7 in Group C and Group H (**g, h**), wound healing on day 14 in Group C and Group H (**i, j**), respectively.

**Table 3:** Intergroup comparisons of WAM values.

	Group C	Group H	Test/p
**t**_0_ **WAM**	12.18/0.81(11.7-13.4)	12.61/0.76(10.5-13.5)	Z_0_= -0.703 *p*=0.482
**t**_1_ **WAM**	10/0.13(9.8-10.5)	9.46/0.66(8.1-10.2)	Z_1_= -1.149 *p*=0.251
**t**_3_ **WAM**	10.34/0.56(9.2-10.9)	7.82/0.82(6.9-8.7)	**Z**_3_**= -2.611** *p*=0.009*
**t**_7_ **WAM**	1.84/0.24(1.3-3.7)	1.64/0.85(1.2-2.9)	Z_7_= -0.731 *p*=0.465
**t**_14_ **WAM**	0.42/1.13(0.2-1.5)	0.05/0.21(0-1.2)	Z_14_= -1.567 *p*=0.117

*: *p*<0,05, Bold p- value indicates statistical significance.

Note: Values reported as medians/IQRs (minimum and maximum). Data were statistically analyzed by the Mann–Whitney U test. Statistical significance was set at p ≤ 0.05.

Abbreviations: WAM, Wound Area Measurement; Group C, control group; Group H, hyaluronic acid group; t0, baseline; t1, day 1; t3, day 3; t7, day 7; t14, day 14.

### Intragroup analysis

HSP 47 values statistically and significantly differed between t0 and t1, t0 and t3, t0 and t7, and t0 and t14 and between t1 and t3, t1 and t7, and t1 and t14 in Group C and Group H (p<0.05). HSP 47 values on t1, t3, t7, and t14 were significantly higher than those at the baseline (t0) and values on t1 were higher than those on t3, t7, and t14 in both groups (p<0.05). No difference was observed between the other time values (t3 and t7, t3 and t14, t7 and t14) (p>0.05).

WAM values at t1, t3, t7, and t14 were significantly lower than those on t0 in Groups C and H (p<0.05). T1 and t3 showed no significant differences regarding WAM values in Group C, whereas the difference was statistically significant in Group H at those intervals. WAM values decreased significantly over time in Group H (p<0.05).

The intragroup analysis of HSP 47 and WAM is shown in Tables 4 and 5, respectively.

**Table 4:** Intragroup analysis of HSP 47 values.

	t_0_	t_1_	t_3_	t_7_	t_14_	Test/p
**Group C**	0.36/0.21(0.2-7.2)	3.47/1.91(0.2-4.2)	0.23/0.09(0.1-0.3)	0.17/0.06(0.1-0.6)	0.18/0.17(0.1-0.3)	Z0−1= −2.023 p=0.043*
						Z0−3= −2.023 p=0.043*
						Z0−7= −2.023 p=0.043*
						Z0−14= −2.023 p=0.043*
						Z1−3= −2.023 p=0.043*
						Z1−7= −2.023 p=0.043*
						Z1−14= −2.023 p=0.043*
						Z3−7= −0.405 p=0.686
						Z3−14= −0.944 p=0.345
						Z7−14= −0.405 p=0.686
**Group H**	0.26/0.13 (0-1.7)	0.27/1.79 (0.2-2.9)	0.16/0.07 (0.1-0.3)	0.13/0.02 (0.1-0.2)	0.18/0.09 (0.1-0.5)	Z0−1=−2.023 p=0.043*
						Z0−3=−2.023 p=0.043*
						Z0−7=−2.023 p=0.043*
						Z0−14=−2.023 p=0.043*
						Z1−3= −2.023 p=0.043*
						Z1−7= −2.023 p=0.043*
						Z1−14= −2.023 p=0.043*
						Z3−7= −0.674 p=0.500
						Z3−14= −1.483 p=0.138
						Z7−14= −1.214 p=0.225

*: *p*<0,05, Bold p- values indicate statistical significance between the related experimental days.

Note: Values reported as medians/IQRs (minimum and maximum). Data were statistically analyzed by the Wilcoxon test. Statistical significance was set at p ≤ 0.05.

Abbreviations: HSP 47, Heat Shock Protein 47; Group C, control group; Group H, hyaluronic acid group; t0, baseline; t1, day 1; t3, day 3; t7, day 7; t14, day 14.

**Table 5:** Intragroup analysis of WAM values.

	t_0_	t_1_	t_3_	t_7_	t_14_	Test/p
**Group C**	12.18/0.81(11.7-13.4)	10/0.13 (9.8-10.5)	10.34/0.56 (9.2-10.9)	1.84/0.24 (1.3-3.7)	0.42/1.13 (0.2-1.5)	Z0−1= −2.023 p=0.043*
						Z0−3= −2.023 p=0.043*
						Z0−7= −2.023 p=0.043*
						Z0−14= −2.023 p=0.043*
						Z1−3= −0.135 p=0.893
						Z1−7= −2.023 p=0.043*
						Z1−14= −2.023 p=0.043*
						Z3−7= −2.023 p=0.043*
						Z3−14= −2.023 p=0.043*
						Z7−14= −2.023 p=0.043*
**Group H**	12.61/0.76(10.5-13.5)	9.46/0.66 (8.1-10.2)	7.82/0.82 (6.9-8.7)	1.64/0.85 (1.2-2.9)	0.05/0.21 (0-1.2)	Z0−1= −2.023 p=0.043*
						Z0−3= −2.023 p=0.043*
						Z0−7= −2.023 p=0.043*
						Z0−14= −2.023 p=0.043*
						Z1−3= −2.023 p=0.043*
						Z1−7= −2.023 p=0.043*
						Z1−14= −2.023 p=0.043*
						Z3−7= −2.023 p=0.043*
						Z3−14= −2.023 p=0.043*
						Z7−14= −2.023 p=0.043*

*: *p*<0,05, Bold p- values indicate statistical significance between the related experimental days.

Note: Values reported as medians/IQRs (minimum and maximum). Data were statistically analyzed by the Wilcoxon test. Statistical significance was set at p ≤ 0.05.

Abbreviations: WAM, Wound Area Measurement; Group C, control group; Group H, hyaluronic acid group; t0, baseline; t1, day 1; t3, day 3; t7, day 7; t14, day 14.

## DISCUSSION

Many alternative agents (such as plant-based remedies) and HA have been used to aid periodontal wound healing. They have been shown to accelerate periodontal wound healing or reduce wound area. However, no agent can be used as an alternative to periodontal treatment or can perform wound healing alone in periodontal treatment.^1-5,20^ In this study, local application of HA decreased gingival wound areas in early wound healing.

HA, a glycosaminoglycan component of the extracellular matrix, is widely used in wound healing because of its biocompatibility and non-immunogenic properties. It supports tissue repair by enhancing fibroblast migration and proliferation and by modulating inflammatory responses by receptors such as RHAMM, ICAM-1, and CD44.^21-25^ Consistent with these findings, HA has also been reported to enhance oral fibroblast activity, increasing their migration capacity by six- to ninefold and promoting proliferation by elevated DNA synthesis within 72 hours. The cross-linked formulation has the advantage of slowing down the degradation rate of HA by hyaluronidase, making it more favorable for clinical applications.^10^ Based on these biological properties, the use of cross-linked HA in this study was preferred as cross-linked and non-cross-linked formulations have been shown to support cell viability and proliferation in oral tissues.

Since no study in the literature is similar to ours, its results were compared with those of studies that applied local HA in isolated wound areas created by punch biopsy.^25^ Similar to our study method, topical HA application on day 7 reduced the wound area created in the palatal region of rats.^25^ Although the results of our study seem compatible with those of that study, WAM on day 3 showed no significant differences from that on day 1 in Group C, whereas WAM on day 3 in Group H was significantly lower than that on day 1. This result shows that locally applied HA can accelerate wound healing from days 1 to 3.

Adjunctive use of HA gel increased healing indices regarding palatal wound healing when compared to chlorhexidine alone on days 3, 7, 14, and 28 compared to the control group.^26^ Healing was completed five days after experimental skin incisions in rats with local application of HA, and it was reported that the large amount of HA in the wound area during healing affected epithelial cell migration, differentiation, and accelerated healing.^27^

In Group H, the decrease in the WAM that was determined in the first three-day period was consistent with Kikuchi et al.,^[28]^ in which they stated that HA plays a role in the synthesis of the matrix elements necessary for cell differentiation in the first phase of wound healing. It has been shown that the treatment can be effective in early wound healing and shorten the healing time.

In this study, HA application resulted in faster healing in the wound areas on day 3 when compared to the control group. On the other hand, the absence of any difference in HSP 47 values suggests that this may be due to other positive effects of HA on the proliferative and migration properties of fibroblasts and its role in increasing vascularization.^10^

Although it has been reported that the positive effect of HA on healing in periodontal tissues involves collagen and cells such as fibroblasts and keratinocytes,^21,22,29^ no study has been found in which its effect on collagen metabolism via HSP 47 was examined. In this study, wound healing after local HA application was evaluated with HSP 47 (a member of the heat shock protein family) and WAM for the first time. Therefore, the HSP 47 results in our study could be compared with those of other studies evaluating HSP 47.

In a study in which HSP 47 was selected as a fibroblast marker in burn healing, the number of HSP 47 positive fibroblasts increased gradually from day 7 to day 14 in the burned skin.^30^ This indicates that HSP 47 increases in the proliferative phase and continues to gradually increase during the proliferative phase. It has been reported that the increase in HSP 47 is proportional to the increased number of fibroblasts and, thus, to improved wound healing.^31,32^ High expression of HSP 47 in the early period of Er:YAG laser application has been stated to indicate accelerated wound healing.^32^ HSP 47 expression has been reported to increase on day 3 in coagulation necrosis induced by CO2 laser irradiation.^33^ Diode laser application increased HSP 47 expression on days 1 and 3 in ulcerated tongue samples.^34^ The significant increase in HSP 47 in day 1 and 3 samples is consistent with the significant increase in HSP 47 on the relevant days in our study. These results show that the HSP 47 increase in early wound healing is proportional to the wound healing rate.

In our study, HA application resulted in no significant difference in HSP47 levels between groups. The absence of any significant intergroup difference in HSP47 values suggests that the positive effects of HA on early gingival wound healing may be more closely related to enhanced cellular migration, proliferation, tissue organization, and modulation of the inflammatory response rather than a direct effect on collagen synthesis via HSP47. However, intragroup analysis showed a significant increase in HSP47 levels when compared to baseline (t0), which is consistent with the findings of Vasques, et al.^34^, who reported an increase in HSP47 during early wound healing.

The decrease in HSP47 levels from days 1 to 3 was greater in our Group C than in Group H. This finding suggests that local HA application may have attenuated the decline in HSP47 levels during this period, potentially exerting a positive effect by supporting collagen production in the early healing. This interpretation is supported by Schimizzi et al., which showed that high-molecular-weight HA gel in a preclinical post-laminectomy rat model changed inflammatory cells, promoting increased cell motility and migration into the wound area.^35^

Moreover, the WAM value on day 3 was not significantly different from the value on day 1 in Group C, whereas the WAM value on day 3 in Group H was significantly lower than the value on day 1. The locally applied HA decreased the wound area from days 1 to 3, and it was seen that it can accelerate wound healing.

The positive outcomes in this experimental rat model regarding HA and HSP 47 expression in wound healing provide a strong foundation for potential translation into human clinical applications. In this study, HA administration was associated with enhanced wound closure, findings consistent with earlier preclinical studies showing its efficacy in accelerating wound closure and improving tissue regeneration.^8,37^ These results are biologically plausible for human application given the conserved role of HA in rat and human skin.

HSP 47 plays a critical role in ensuring proper folding and stability of procollagen molecules within the endoplasmic reticulum. Since no significant differences were observed between the groups, a marked decrease was noted in Group C, whereas HSP 47 levels remained more stable in the Group H. Although the differences were statistically insignificant on the specified days, HA seems to mitigate the decrease in Group H. These findings suggest that modulating collagen-related pathways, including HSP47 activity, may have translational relevance in wound healing disorders characterized by impaired or excessive collagen production, such as chronic wounds and fibrotic scarring.^38^ In this context, HA may represent a promising adjunctive therapeutic agent for improving tissue repair and wound healing outcomes. Its incorporation into novel wound care products or adjunct therapies may enhance healing rates, improve scar quality, and address unmet needs in chronic or complex wound management.

### Study limitations

This research had several strengths, including the fact that it was the first study in which the effect of HA on gingival wound healing was examined in conjunction with HSP 47 and clinical WAM. However, it also has limitations, such as its evaluation of the anti-inflammatory and antioxidant properties of HA together with its effect on collagen metabolism. A limitation of this study refers to its absence of a placebo or vehicle control group. Therefore, we are unable to completely exclude that a portion of the observed effect may be related to the gel base rather than HA. Future studies including a vehicle control group are warranted to clarify this distinction. The effect of HA on fibroblast migration and proliferation ability should be evaluated with the change in HSP 47 amounts. Another limitation is the use of female rats. Hormonal factors, particularly estrogen, may influence inflammatory response and collagen synthesis, potentially affecting wound healing dynamics and HSP 47 expression.

Future investigations should focus on optimizing HA dosage, evaluating the long-term effects of HSP 47 modulation, and developing delivery systems such as bioengineered scaffolds, hydrogels, or controlled-release dressings to improve therapeutic efficacy. Wound healing was evaluated only in its early stages (days 0–14). Long-term outcomes, including scar formation and tissue remodeling, were ignored, and should be considered in future studies. Since HSP 47 is closely associated with collagen synthesis, studies combining biochemical and histological analyses would more comprehensively understand the underlying mechanisms.

## CONCLUSION

The findings in this study indicate that topical high–molecular-weight hyaluronic acid enhances early gingival wound healing in a rat model. Although HA did not significantly alter HSP47 levels between groups, it may support early wound healing by modulating cellular responses and tissue reorganization.

## Data Availability

The datasets used and analyzed during this study are available from the corresponding author upon reasonable request.
